# Detecting carbapenem-resistant *Acinetobacter baumannii* (CRAB) carriage: Which body site should be cultured?

**DOI:** 10.1017/ice.2020.197

**Published:** 2020-08

**Authors:** Amir Nutman, Elizabeth Temkin, Jonathan Lellouche, Debby Ben David, David Schwartz, Yehuda Carmeli

**Affiliations:** 1 National Institute for Antibiotic Resistance and Infection Control, Ministry of Health, Tel-Aviv Sourasky Medical Center, Tel-Aviv, Israel; 2 Sackler Faculty of Medicine, Tel-Aviv University, Tel-Aviv, Israel

## Abstract

We compared the yield of culturing various body sites to detect carriage of carbapenem-resistant *Acinetobacter baumannii* (CRAB). Culturing the skin using a premoistened sponge, with overnight enrichment and plating on CHROMagar MDR *Acinetobacter*, had the highest yield: 92%. Skin is satisfactory as a single site for active surveillance of CRAB.

*Acinetobacter baumannii* is a multidrug-resistant pathogen causing severe infections in hospitals and long-term care facilities. Detecting carriers is a mainstay of controlling nosocomial spread of resistant organisms. Screening for carbapenem-resistant *Acinetobacter baumannii* (CRAB) has been recommended to control outbreaks, but no specific recommendations have been made regarding which body sites to culture.^1^ In 2007, we studied the yield of culturing the nose, throat, axilla, groin, rectum, open wounds, and tracheal aspirates, and no site or combination of sites had high enough sensitivity to be recommended for CRAB screening.^2^ Since then, improved culture media^3^ and a skin sampling technique using a premoistened sponge^4^ have increased test sensitivity. Recently, we used these methods on patients with clinical cultures positive for CRAB; compared to that gold standard, screening cultures from the buccal mucosa, skin, and the rectum combined achieved 94% sensitivity.^5^ That study was too small to determine whether screening a single body site is sensitive enough to detect carriers and which body site should be chosen. Here we summarize our cumulative experience of screening for CRAB carriage as an evidence base to assist in developing guidelines for CRAB screening.

## Methods

### Study setting and patients

In 2015–2019, patients were screened for CRAB as part of an ongoing infection control program in 2 settings: adult wards at a tertiary- acute-care hospital (ACH) and a chronic ventilation ward at a post–acute-care hospital (PACH). Chlorhexidine bathing is routinely performed at both institutions.

The primary outcome was screening yield for each body site sampled. Because there is no gold standard for CRAB screening, a patient was defined as a CRAB carrier if a culture from any of the sites sampled was positive.

### Specimen collection methods

Buccal mucosa and rectal specimens were collected using swabs (Amies agar gel transport swab; Copan Italia S.P.A., Brescia, Italy). Tracheal aspirates were collected from ventilated patients using a suction catheter. Sponges premoistened with a phosphate buffer (Polywipe; Medical Wire & Equipment, Wiltshire, England) were used to sample the skin by swiping down both arms and legs from top to bottom (1 sponge for all 4 limbs).

### Microbiological methods

Specimens were inoculated, after overnight enrichment in brain-heart infusion (BHI) broth (Hylabs, Rehovot, Israel), onto CHROMagar MDR *Acinetobacter* plates (Hylabs, Rehovot, Israel), and incubated overnight at 37ºC. Suspicious colonies were identified to the species level using VITEK-MS (bioMérieux, Marcy l’Etoile, France) followed by *bla*_OXA-51-like_ gene PCR. Carbapenem resistance was determined using VITEK-2 (bioMérieux, Marcy l’Etoile, France).

### Statistical analysis

We compared yield by body site and by setting using a test of proportions. Analyses were done using Stata version 14.2 software (StataCorp, College Station, TX).

## Results

The sample consisted of 612 specimens from 201 patients who tested positive for CRAB in at least 1 body site: 100 from the ACH and 101 from the PACH. The yields from single body sites and combinations of body sites are presented in Table 1. The site with the highest yield was the skin (91.9%; 95% confidence interval [CI], 87%–95%), followed by the buccal mucosa (62.5%; 95% CI, 54%–71%). The yield was low for tracheal aspirate (49.1%; 95% CI, 39%–59%) and the rectum (47.3%; 95% CI, 40%–55%). Skin sampling had similar yield in the ACH (91.0%) and PACH (92.8%; *P* = .64) settings. The yield from buccal mucosa was higher in the ACH (68.7%) than in the PACH (45.9%; *P* = .01); the yield from the rectum was also higher in the ACH, but not significantly so (54.3% vs 42.2%; *P* = .13). Only the combination of skin and buccal mucosa sampling had a significantly higher yield than skin alone (*P* = .003). In carriers with a negative skin culture, the buccal mucosa cultures were positive in 9 of 10 of these carriers (90%), the rectal samples in 2 of 4 carriers (50%), and tracheal aspirates in 2 of 7 carriers (28.6%).

**Table 1.** CRAB Screening Yield Among 201 Patients Positive for CRAB by Body Site

Body Site	No. Sampled	No. Positive	Yield, % (95% CI)
Buccal mucosa	136	85	62.5 (54–71)
Tracheal aspirate	110	54	49.1 (39–59)
Skin	197	181	91.9 (87–95)
Rectum	169	80	47.3 (40–55)
Buccal mucosa + skin	136	135	99.3 (96–100)
Buccal mucosa + rectum	107	74	69.2 (59–78)
Skin + rectum	165	159	96.4 (92–99)
Sputum + rectum	99	62	62.6 (52–72)
Sputum + skin	106	101	95.3 (89–98)

Note. CRAB, carbapenem-resistant *A. baumannii*; CI, confidence interval.

## Discussion

Culturing the skin using a premoistened sponge had a sensitivity of 92% to detect CRAB carriage. All other screening sites had low sensitivity; even combinations of 2 sites (excluding the skin) reached a maximum of 69% sensitivity. The combination of buccal mucosa and skin was 99% sensitive. Tracheal aspirate, often used to screen for CRAB carriage in ventilated patients,^6^ had a low yield in our study (49%). The yield from skin screening was similar at the 2 study centers, suggesting that it is less influenced by sampling technique or patient characteristics. These results, combined with those of previous studies,^4,5^ suggest that the skin is the main colonization site for CRAB and that it is usually sparse on the skin. Thus, a large surface area is required for sampling using a highly absorbent sponge and enrichment for detection.

Identifying CRAB carriers can direct various infection control activities, including isolation and cohorting of carriers, environmental cleaning, decolonization (eg, chlorhexidine bathing), and removing contact precautions when carriage has resolved. Detection of CRAB carriage can also assist in empiric therapy choice in the event of infection.

The sensitivity of skin screening for CRAB observed in our study (92%) compares favorably with the sensitivity of screening methods commonly used for other multidrug-resistant organisms. For example, culturing the nares for MRSA has up to 90% sensitivity.^7^ Screening by rectal swab or stool specimen has 61%–99% sensitivity to detect VRE,^8^ and 76%–100% sensitivity to detect CRE.^9^

An important issue to consider before implementing a screening policy is the pretest probability of screening positive. When the prevalence in the screened population is <10%, <1 positive patient will be missed for every 100 patients screened using a test with 90% sensitivity. When prevalence is higher or when missing a case may have severe consequences, a test with a higher sensitivity should be chosen. Adding a buccal mucosa swab to skin sampling increased sensitivity from 92% to 99%; thus, screening both sites may be preferred in such circumstances.

In a previous study, CRAB active surveillance by rectal swabs and bronchial aspirates using a rapid molecular diagnostic assay that provided results “within a few hours after sample collection,” followed by prompt isolation of carriers, decreased CRAB acquisition by 35% compared to conventional cultures.^10^ Our methods, although sensitive, are not rapid; they provide a result of “suspected CRAB” in 36 hours and a final result after identification and susceptibility testing, which require an additional 24 hours.

This study has several limitations. Since there is no gold standard for screening, we measured sensitivity at each body site by comparing it to a standard of positivity at any body site. This may be an underestimation, in which case the sensitivities we calculated may be inflated. Also, our study was performed in only 2 centers.

In conclusion, our data support the notion that the skin is the main site of colonization by CRAB, and that culturing the skin using a premoistened sponge as a single site for active surveillance of CRAB is satisfactory. The sensitivity of screening the buccal mucosa, rectum, and tracheal aspirate was low, and a negative screening culture from these sites should not be taken as evidence of CRAB noncarriage.

## References

[1] Tacconelli E, Cataldo MA, Dancer SJ, et al. ESCMID guidelines for the management of the infection control measures to reduce transmission of multidrug-resistant gram-negative bacteria in hospitalized patients. Clin Microbiol Infect 2014;20 suppl 1:1–55. 10.1111/1469-0691.12427 24329732

[2] Marchaim D, Navon-Venezia S, Schwartz D, et al. Surveillance cultures and duration of carriage of multidrug-resistant *Acinetobacter baumannii*. J Clin Microbiol 2007;45:1551–1555. 17314222 10.1128/JCM.02424-06 PMC1865886

[3] Moran-Gilad J, Adler A, Schwartz D, Navon-Venezia S, Carmeli Y. Laboratory evaluation of different agar media for isolation of carbapenem-resistant *Acinetobacter* spp. Eur J Clin Microbiol Infect Dis 2014;33:1909–1913. 24865248 10.1007/s10096-014-2159-y

[4] Doi Y, Onuoha EO, Adams-Haduch JM, et al. Screening for *Acinetobacter baumannii* colonization by use of sponges. J Clin Microbiol 2011;49:154–158. 20980559 10.1128/JCM.01043-10 PMC3020416

[5] Nutman A, Lerner A, Schwartz D, Carmeli Y. Evaluation of carriage and environmental contamination by carbapenem-resistant *Acinetobacter baumannii*. Clin Microbiol Infect 2016;22:949.e5–949.e7. 10.1016/j.cmi.2016.08.020 27596532

[6] Rodríguez-Baño J, García L, Ramírez E, et al. Long-term control of hospital-wide, endemic multidrug-resistant *Acinetobacter baumannii* through a comprehensive “bundle” approach. Am J Infect Control 2009;37:715–722. 19457584 10.1016/j.ajic.2009.01.008 PMC2783564

[7] Safdar N, Narans L, Gordon B, Maki DG. Comparison of culture screening methods for detection of nasal carriage of methicillin-resistant *Staphylococcus aureus*: a prospective study comparing 32 methods. J Clin Microbiol 2003;41:3163–3166. 12843058 10.1128/JCM.41.7.3163-3166.2003 PMC165368

[8] Faron ML, Ledeboer NA, Buchan BW. Resistance mechanisms, epidemiology, and approaches to screening for vancomycin-resistant *Enterococcus* in the health care setting. J Clin Microbiol 2016;54:2436–2447. 27147728 10.1128/JCM.00211-16 PMC5035425

[9] Adler A, Navon-Venezia S, Moran-Gilad J, Marcos E, Schwartz D, Carmeli Y. Laboratory and clinical evaluation of screening agar plates for detection of carbapenem-resistant Enterobacteriaceae from surveillance rectal swabs. J Clin Microbiol 2011;49:2239–2242. 21471338 10.1128/JCM.02566-10 PMC3122751

[10] Yamamoto N, Hamaguchi S, Akeda Y, et al. Rapid screening and early precautions for carbapenem-resistant *Acinetobacter baumannii* carriers decreased nosocomial transmission in hospital settings: a quasi-experimental study. Antimicrob Resist Infect Control 2019;8:110. 31297191 10.1186/s13756-019-0564-9 PMC6598269

